# Severe acute respiratory syndrome coronavirus 2 viral load in respiratory and feces specimens of children with coronavirus disease 2019

**DOI:** 10.2217/fvl-2020-0180

**Published:** 2021-01-26

**Authors:** Xiang Ma, Liang Su, Lu Cheng, Zhaohua Zhang, Jing Sun, Miao Liu, Jing Wang, Xuemei Jiang, Yun Zhang, Yuling Han, Zhongfa Zhang, Zhongtao Gai

**Affiliations:** 1 ^1^Department of Respiratory, Qilu Children’s Hospital, Cheeloo College of Medicine, Shandong University, Jinan, Shandong 250022, China; 2 ^2^Jinan Infectious diseases Hospital of Shandong University, Jinan, Shandong 250021, China; 3 ^3^Jinan Institute of Pediatrics, Qilu Children’s Hospital, Cheeloo College of Medicine, Shandong University, Jinan, Shandong 250022, China

**Keywords:** children, coronavirus, COVID-19, severe acute respiratory syndrome coronavirus 2 (SARS-CoV-2), viral load

## Abstract

**Background:** The viral load kinetics of children with coronavirus disease 2019 is not clear. **Materials & methods:** The viral load of throat, nasal and feces specimens of 10 children with coronavirus disease 2019 were detected and analyzed. **Results:** The virus load of nasal and throat specimen decreased extremely and all respiratory specimens tested negative on the third week after they were admitted. All children showed positive PCR results in their feces. A total of 70% children showed positive results at the fourth week and 40% children showed positive results in their feces at the fifth week. All children tested negative on the sixth week. **Conclusion:** The positive rate of stool in children was higher than that in adults and the shedding time of stool was longer than that of respiratory specimen.

Coronavirus disease 2019 (COVID-19), which is caused by severe acute respiratory syndrome coronavirus 2 (SARS-CoV-2), has an extremely high global level risk by the WHO. Unfortunately, more than 25 million people have been infected with the virus to date, and 800,000 patients died [1]. While the information on epidemiologic investigation and clinical manifestation are accumulating, the viral kinetics of the novel virus have not been systematically evaluated yet, especially in children. Given that children’s clinical characteristics are different from adults, most children have mild or asymptomatic infections; their viral load and viral shedding pattern and persist time also differ from adults in several reports [2–5]. The behavior of the virus in children should be understood. Thus, we present the viral load kinetics of 10 children in Jinan, China to assess the virus’ activity pattern and prompt people to take improved protective measures toward this age range.

## Materials & methods

### Patients

From 25 January 2020 to 10 March 2020, 10 children were admitted in Jinan Infectious Diseases Hospital with COVID-19 in Jinan, Shandong Province, China. Patients were followed up regularly after discharge until 25 March 2020, which was the final date of follow-up. We monitored SARS-CoV-2 viral loads in respiratory and stool specimens that were obtained from 10 children (seven girls and three boys, median age: 4.7 years old; range: 11 months to 14 years and 9 months). These children all had close contacts with their families who were confirmed to be infected by the virus and were admitted to the hospital on the day or the day after they obtained a positive SARS-CoV-2 nucleic acid results of their respiratory specimen.

### Sample collection & detection of SARS-CoV-2

We analyzed the viral load in nasal/throat specimen that was obtained from the 10 children every 3–5 days after they were admitted in the hospital. During the routine follow-up of seven children who were discharged from the hospital, their stools tested positive. Then, we conducted stool tests for all children until all stool specimens showed negative results [4]. SARS-CoV-2 (ORF1ab/N) nucleic acid detection kit (Bio-germ, Shanghai, China) was used to detect the presence of SARS-CoV-2 RNA by real-time fluorescence reverse transcription polymerase chain reaction (RT-PCR). Total RNA was extracted using TRIzol according to the previous instructions [6]. RT-PCR was performed using the 2019-nCoV (ORF1ab/N) nucleic acid detection kit (Bio-germ) according to the manufacturer’s instructions. The following primers and probe for CoV envelope genes were used: forward primer: 5’-TCAGAATGCCAATCTCCCCAAC-3’, reverse primer: 5’-AAAGGTCCACCCGATACATTGA-3’ and probe: 5’-CY5-CTAGTTACACTAGCCATCCTTACTGC-3’ BHQ1. The amplification conditions were 50°C for 15 min and 95°C for 3 min, followed by 45 cycles of 95°C for 15 s and 60°C for 30 s.

A Ct of less than 40 with a clear amplification curve was considered a positive test and a value of more than 40 indicated that the virus is not molecularly detectable. Meanwhile, if these patients do not have any clinical symptom and the Ct value is extremely close to or above 40, then we will stop the test to reduce the child’s discomfort. The Ct value is used to reflect the viral load in the respiratory specimens roughly (inversely proportional to the Ct value) [7]. All operations were performed by professionals.

### Ethics

This study was conducted in accordance with the Declaration of Helsinki. It was approved by the ethical committee in Jinan Infectious Diseases Hospital (2020-JC-33). All the children’ parents signed informed consent forms.

## Results

Among these 10 patients, four children had symptoms such as fever (cases 2, 3 and 10; 3/10, 30%) and cough (case 5, 1/10, 10%). The six other children (60%) were asymptomatic. Case 1 was a young girl with no symptoms. After 15 days in the hospital, she was discharged with her parents. However, 2 weeks after discharge from the hospital, case 1’s stool test at home showed that she was positive with SARS-CoV-2. Thus, this patient was admitted to the hospital again and her stool nucleic acid was tested extremely late (Table 1). Case 4 was not followed up because the last test of cycle threshold (Ct) was close to 40 or higher than 40 cycles.

**Table 1.** Viral load of different specimen of children with coronavirus disease 2019 (Ct value).

	Case1			Case2			Case3			Case4			Case5			Case6			Case7			Case8			Case9			Case10		
	NS	TS	SS	NS	TS	SS	NS	TS	SS	NS	TS	SS	NS	TS	SS	NS	TS	SS	NS	TS	SS	NS	TS	SS	NS	TS	SS	NS	TS	SS
1–7 days	28.34	26.47		23.8	24.65		32.4	31.91		23.98	29.87		34.23	32.11		32.48	31.68		30.54	30.99		32.24	32.92		29.88	30.85		35.15	36.26	
8–14 days	31.91	37.24		36.7	37.23		37.61	41.24		41.92	39.11		37.35	35.45		33.49	35.76		39.45	40.61		35.67	35.61		38.63	39.2		37.22	37.22	
15–21 days	42.45	42.48		42.44	41.34		42.38	42.66					42.12	42.48		39.72	38.86				33.45			39.13			27.11			33.42
22–28 days						35.43			37.06			33.76			38.51			32.13			37.8			41.34			31.6			42.22
29–35 days			37.06			36.24			41.45			34.11			42.38			32.89			42.22			41.6			40.19			
36–42 days			42.58			41.54												42.0												

All the tests and results are counted from admission day.

NS: Nasal swab; SS: Stool specimen; TS: Throat swab.

**Figure 1.**
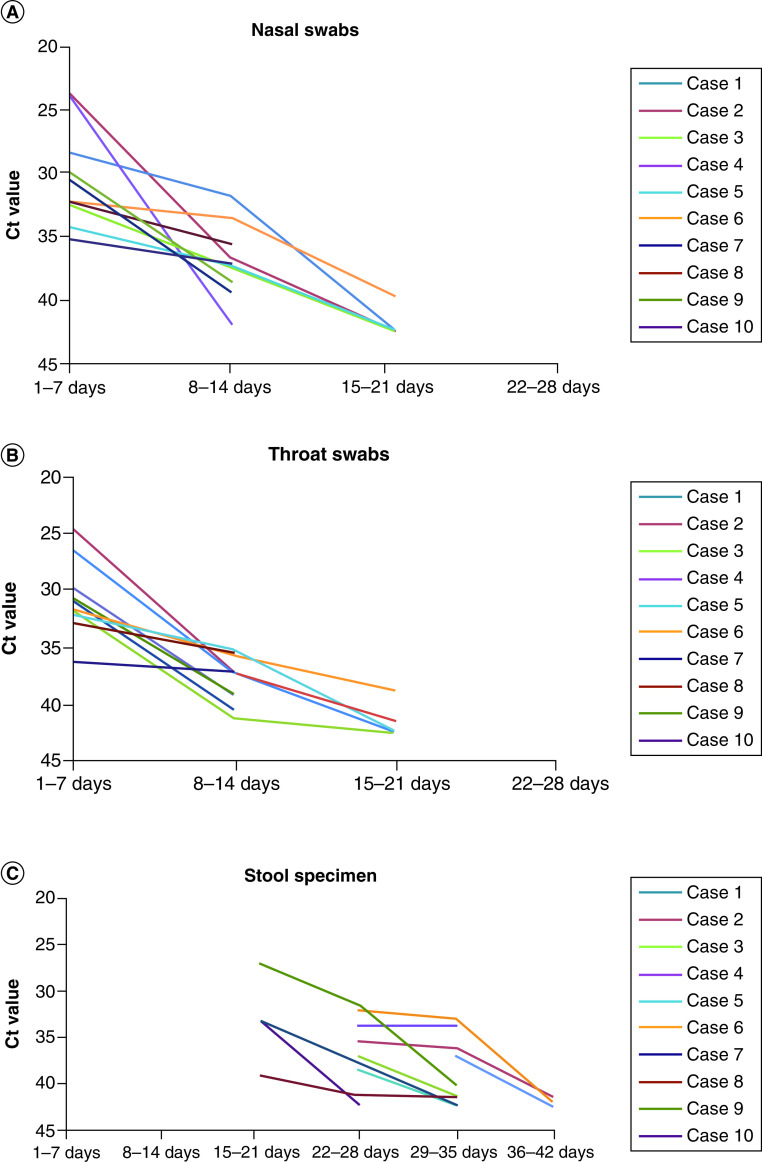
Variation trend of nucleic acid detection results of different specimen. **(A)** Variation trend of nucleic acid detection results of nasal swabs. **(B)** Variation trend of nucleic acid detection results of throat swabs. **(C)** Variation trend of nucleic acid detection results of sto**ol** specimen. All tests and results were counted from the admission day of the patients.

A total of 25 nasal swabs (sampled from the mid-turbinate and nasopharynx, Figure 1A), 25 throat swabs (Figure 1B) and 25 stool specimens were analyzed (Figure 1C, all results are shown in Table 1).

Figure 1A and B show that the virus loads of nasal and throat specimens decreased rapidly, respectively. On the second week, two patients’ throat swabs tested negative and one patient’s nasal swab tested negative. Meanwhile, on the third week, all patients’ nasal and throat swabs tested negative. However, Figure 1C shows that seven children showed positive results (70%) in their stool specimen on the fourth week. Even on the fifth week, four children still showed positive results (40%) in their feces and all children tested negative on the sixth week (36–42 days).

## Conclusion & future perspective

This study revealed the change trend in the viral load of respiratory and gastrointestinal specimens in 10 children with COVID-19. At the shortest, the observed duration of viral shedding among survivors was 8 days, whereas the longest was 37 days in patients from Wuhan, China. The virus was continuously detectable until death in nonsurvivors. Meanwhile, the differences between severe (19 days) and critical patients in a previous study are not significant (24 days) [8]. A previous study also reported that the virus shedding time even reaches up to 49 days in certain individuals [9]. In the present study, all pediatric patients’ respiratory tract specimens tested negative on the third week or even earlier, which was similar to results of most previous studies [10–12]. However, several studies found a higher viral load in sputum specimens than in nasal and throat swabs [13] and various works have found a higher viral load in nasal swabs than in throat swabs [12]. However, the difference between the viral load in nasal swabs and throat swabs in our study was not significant. Whether this phenomenon differs from children and adults still needs additional in-depth research. Given our relatively small number of cases, we cannot draw strong conclusions for this phenomenon.

At present, SARS-CoV-2 uses angiotensin-converting enzyme 2 as an entry receptor [14]. Studies on SARS-CoV-2 have shown that angiotensin-converting enzyme 2 is highly expressed in esophageal epithelial cells and absorptive enterocytes from ileum and colon, thereby suggesting possible fecal transmission [15]. Several studies showed that patients with COVID have digest symptoms, such as diarrhea, vomiting, nausea and abdominal pain and live viruses or viral nucleic acids can be detected in feces [16–20]. Further research showed that 36–53% fecal PCR becomes positive 2–5 days later than patients who are sputum PCR positive. Fecal excretion persists after sputum excretion in 23–82% patients for 1–11 days [19]. In the present study, stool samples were positive in all children (100%), although they did not have any digestive symptom, which was much higher than that reported in adults [19]. At the same time, the shedding time of stool sample was much longer than respiratory specimens, that is, reaching even up to more than 5 weeks. Our team also found that children have longer shedding time than adults in feces sample [4]. Therefore, given that many children are asymptomatic or mildly infected [2,3], they have a high proportion of stool poisoning and long shedding time. Therefore, once they are infected, children will become hidden and have the a high risk of transmission.

In conclusion, the results of this study suggested that virus shed longer in the gastrointestinal tract than in the respiratory tract in children. A study has shown that viable virus existed for at least 3 h in aerosols and 2 or 3 days on plastic and stainless steel surfaces [21]. According to the high viral infectivity of SARS-CoV-2, long shedding time in feces and poor hand hygiene in children, we believe that exposure to a fecal-contaminated environment may cause the fecal–oral route of transmission. The virus may infect their healthy families, classmates and friends through the fecal–aerosol–respiratory pathway by sharing toilets.

Children are going back to school in many places in China. For children who do not good hand hygiene, the problem of avoiding cross-infection among children, especially cross-infection caused by feces, should be considered by public health personnel and regulations should be strictly implemented. The latest management guide in China included discharging patients from the hospital after two RT-PCR negative tests of respiratory specimens were carried out with more than 24 h of interval and that the patient should be isolated for 14 days after discharge [22]. The stool samples of the discharged patient could still be positive, especially in children [4]. Hence, the discharge criteria should be carefully evaluated, especially among children. We also recommend a test for fecal nucleic acid before a patient is released from isolation.

Our report presented a number of limitations. Given that we only presented 10 children with mild or asymptomatic infection, the data obtained may not be generalizable to all cases, especially severe ones. Second, we tested the feces sample after 2 weeks and test respiratory specimen every 3–5 days, we cannot evaluate the exact change trend for all patients. Third, we cannot estimate the exact time at which the children were exposed to the virus because they were all diagnosed after close contact with their families that were confirmed to be infected with the virus. Fourth, given that positive RNA testing is not a proxy for infectivity, this study showed that stool positivity is a problem and that other infectious factors are needed to determine whether prevention and control measures, such as the isolation of live viruses or discovery of evidence of transmission, are needed. Appropriate precautions should be taken for any positive result. Further studies are needed to understand SARS-CoV-2 infection. Infection control measures should be reviewed with the rapidly evolving epidemiology of COVID-19 and important questions we are about to face, that is, children go back to school.

**Summary points**

- The study presented the viral load of severe acute respiratory syndrome coronavirus 2 of different specimens in children with coronavirus disease 2019.
- All the stool specimens of the children with coronavirus disease 2019 tested positive for severe acute respiratory syndrome coronavirus 2 RNA.
- The shedding time of children’s stool was longer than that of the respiratory specimen.
- We should be concerned about the changes in fecal RNA in children and prevent fecal–oral transmission.
